# An Efficient Ice Sheet/Earth System Model Spin‐up Procedure for CESM2‐CISM2: Description, Evaluation, and Broader Applicability

**DOI:** 10.1029/2019MS001984

**Published:** 2020-08-22

**Authors:** Marcus Lofverstrom, Jeremy G. Fyke, Katherine Thayer‐Calder, Laura Muntjewerf, Miren Vizcaino, William J. Sacks, William H. Lipscomb, Bette L. Otto‐Bliesner, Sarah L. Bradley

**Affiliations:** ^1^ Department of Geosciences University of Arizona Tucson AZ USA; ^2^ Climate and Global Dynamics Laboratory National Center for Atmospheric Research Boulder CO USA; ^3^ Associated Engineering Group Ltd. Calgary Alberta Canada; ^4^ Department of Atmospheric and Oceanic Sciences University of Colorado Boulder Boulder CO USA; ^5^ Department of Geoscience and Remote Sensing Delft University of Technology Delft Netherlands; ^6^ Department of Geography University of Sheffield Sheffield UK

**Keywords:** Community Earth System Model version 2 (CESM2), Coupled ice‐sheet/Earth system modeling, Interactive ice sheets, Spin‐up simulation

## Abstract

Spinning up a highly complex, coupled Earth system model (ESM) is a time consuming and computationally demanding exercise. For models with interactive ice sheet components, this becomes a major challenge, as ice sheets are sensitive to bidirectional feedback processes and equilibrate over glacial timescales of up to many millennia. This work describes and demonstrates a computationally tractable, iterative procedure for spinning up a contemporary, highly complex ESM that includes an interactive ice sheet component. The procedure alternates between a computationally expensive coupled configuration and a computationally cheaper configuration where the atmospheric component is replaced by a data model. By periodically regenerating atmospheric forcing consistent with the coupled system, the data atmosphere remains adequately constrained to ensure that the broader model state evolves realistically. The applicability of the method is demonstrated by spinning up the preindustrial climate in the Community Earth System Model Version 2 (CESM2), coupled to the Community Ice Sheet Model Version 2 (CISM2) over Greenland. The equilibrium climate state is similar to the control climate from a coupled simulation with a prescribed Greenland ice sheet, indicating that the iterative procedure is consistent with a traditional spin‐up approach without interactive ice sheets. These results suggest that the iterative method presented here provides a faster and computationally cheaper method for spinning up a highly complex ESM, with or without interactive ice sheet components. The method described here has been used to develop the climate/ice sheet initial conditions for transient, ice sheet‐enabled simulations with CESM2‐CISM2 in the Coupled Model Intercomparison Project Phase 6 (CMIP6).

## Key Points

- We describe a computationally tractable, iterative procedure for spinning up a coupled Earth system‐ice sheet model
- Equilibrium state from the iterative procedure is similar to a more expensive traditional model spin‐up with prescribed ice sheets
- The procedure is used for developing initial conditions for transient, fully coupled simulations in the Coupled Model Intercomparison Project phase 6

## 1 Introduction

Continental‐scale ice sheets are integral parts of the Earth system, which directly and indirectly interact with other components of the Earth system over a range of different time scales; see the recent review by Fyke et al. (2018). At present there are two ice sheets on the planet: the Antarctic ice sheet (AIS; ice volume around 58 m global mean sea level equivalent; Fretwell et al., 2013) in the Southern Hemisphere and the Greenland ice sheet (GrIS; ice volume around 7 m global mean sea level equivalent; Morlighem et al., 2017) in the Northern Hemisphere. These ice sheets are potentially highly susceptible to anthropogenic climate change, which is enhanced at high latitudes (so‐called *polar amplification*) by internal feedback processes in the climate system (e.g., Graversen et al., 2008; Serreze & Francis, 2006; Smith et al., 2019). Satellite‐based monitoring programs indicate that both the GrIS and AIS have been losing mass at an accelerated rate in the last few decades and they are currently contributing about 1 mm to global sea level rise each year (Rignot & Kanagaratnam, 2006; Rignot et al., 2019; Van den Broeke et al., 2016). Understanding the bidirectional climate‐ice sheet coupling and sensitivity is thus a high priority for ongoing and future research that aims to improve sea level rise projections, impact assessments, and adaptation planning (Fyke et al., 2018).

The history of coupled climate‐ice sheet modeling dates back several decades. However, because of computational limitations exploring coupled climate‐ice sheet interactions over glacial timescales, model development and research to date have primarily been limited to computationally efficient intermediate‐complexity models, often relying on simplified climate, mass balance, and ice‐flow calculations (e.g., Bauer & Ganopolski, 2017; Calov et al., 2015; Charbit et al., 2013; Ganopolski et al., 2010; Liakka et al., 2011; Robinson et al., 2011; Roe & Lindzen, 2001). Conversely, highly complex Earth system models (ESMs), such as those taking part in Coupled Model Intercomparison Project (CMIP) cycles, have traditionally represented ice sheets as prescribed (or passive) “white mountains,” which interact with the circulation through topography, albedo, and surface snow effects but do not themselves respond to the simulated climate.

Recently, several major modeling centers have started incorporating interactive thermo‐mechanical ice sheet components into their ESMs—notably, models participating in the Ice Sheet Model Intercomparison Project (ISMIP6) (Nowicki et al., 2016) under the auspice of the CMIP6 (Eyring et al., 2016). The newly released Community Earth System Model, Version 2 (CESM2) (Danabasoglu et al., 2020) is one such highly complex ESM within ISMIP6 to include interactive (or two‐way) coupling between a dynamic ice sheet model of the GrIS (Lipscomb et al., 2019) and the broader ESM.

Climate model simulations of historical (nominally, year 1850 CE to present) and future (e.g., to year 2100, as in CMIP6 standard simulations) require an appropriate, physically consistent initial global coupled climate condition from which to start. This initial condition should be free of any residual drift related to the numerical equilibration of simulated climate components, both in terms of their internal states and in terms of fluxes between components. Development of such initial conditions represents a major task that is common to all modeling centers and typically requires a substantial fraction of each center's computing resources. Including an interactive ice sheet component into global coupled model frameworks adds considerable complexity to coupled model spin‐up procedures.

A primary challenge arises because ice sheets have large thermal and dynamic inertia and thus respond slowly to imposed climate forcing at the whole‐ice sheet scale, notwithstanding dynamic processes at the ice sheet margins and in ice streams that can respond comparatively quickly. Standalone ice sheet model simulations of the GrIS typically reach steady state in the order of 10,000 years when run under a constant forcing protocol (e.g., Koenig et al., 2015; Stone et al., 2010). This is even longer than the equilibrium timescales of deep ocean biogeochemical tracers and the soil carbon and nitrogen pools (Ilyina et al., 2013; Thornton & Rosenbloom, 2005), which traditionally have been the primary equilibration bottlenecks when spinning up ESMs. An important consequence of multimillennial ice sheet inertia is that the observed GrIS is not in perfect balance with recent Holocene climate and is still adjusting from intermediate‐ and long‐term climate shifts such as the “Little Ice Age” (e.g., Fischer et al., 1998) and seasonal variations in insolation from long‐term changes in Earth's orbital configuration (e.g., Vinther et al., 2009). Ideally, residual GrIS drift should be accounted for in the course of obtaining 1850 CE initial conditions, in order to obtain more realistic initial trends in area, volume, and mass balance that carry forward in a physically realistic manner to present‐day simulated GrIS changes.

Second, ice sheets are sensitive to both direct and indirect bidirectional ice sheet/Earth system feedback processes (Fyke et al., 2018) that can strongly influence their growth trajectories, height, extent, and morphology (e.g., DeConto et al., 2007; Ridley et al., 2005). Feedbacks can be complex, involve multiple climate components, and generate complicated and counterintuitive behavior; for example, ice growth in one region can result in stagnation or even retreat in other areas from feedback‐induced changes to local climate conditions (see paleo‐climate examples in Lofverstrom et al., 2015; Lofverstrom & Liakka, 2016, 2018; Tulenko et al., 2020). A consequence of ice sheet/Earth system coupling is that uncoupled methods, such as standalone ice sheet model simulations, inverse methods, and data assimilation techniques (e.g., Aschwanden et al., 2019; Goelzer et al., 2017; Lee et al., 2015), tend to struggle when applied to the generation of self‐consistent ice sheet states within coupled climate models. This is because spurious transients—both in the ice sheet and in the other model components—emerge when an externally spun‐up ice sheet instance is introduced into a coupled model that it has not had a previous chance to equilibrate with.

Given the above‐mentioned physical characteristics of the coupled ice sheet/climate system, at present an ideal coupled ice sheet/climate model spin‐up would entail a transient, coupled ice sheet/climate simulation over the entire Last Glacial Cycle. Although this would capture ice sheet/climate feedback processes and long whole‐ice sheet response times, it is computationally infeasible to run a highly complex ESM for more than a few centuries (see Tables 1 and 2), precluding a full glacial cycle simulation in reality. Recognizing this challenge, one method to reduce the computational cost is to accelerate the ice sheet (e.g., asynchronous or periodic synchronous coupling) with respect to the other model components (e.g., Mikolajewicz et al., 2007; Ridley et al., 2005; Ziemen et al., 2019). This approach leverages the fact that year‐to‐year changes in ice sheet area and topography are typically small, even at the margins where the ice sheet is highly dynamic with fast outlet glaciers and pronounced summer ablation. Accelerating the ice sheet component is thus an attractive solution to reduce the overall integration length of coupled models. However, pure asynchronous coupling techniques introduce inconsistencies in the overall climate evolution and should be used cautiously and only if the model climate is close to equilibrium; that is, when the long‐term climate drift is small compared to interannual variability. Moreover, even with moderate acceleration factors (Ganopolski et al., 2010), simple asynchronous acceleration can still be computationally infeasible for highly complex coupled models (Table 2), especially for repeated simulations in sensitivity or perturbed initial condition ensembles.

**Table 1:** Comparison of Model Cost (Core Hours per Simulated Year), Throughput (Simulated Years per Wall‐Clock Day), and Total Processor Count for the Different Model Configurations Used in the Spin‐up Procedure

Model configuration	Fully coupled	Data atmosphere	Ice‐sheet only
Cost	2,800	900	0.3
Throughput	18	75	23,000
Total CPUs	2,160	2,772	288
Optimized layouts			
Coupler	1,800 (0)	1,044 (0)	288 (0)
Atmosphere	1,800 (0)	36 (1,008)	288 (0)
Land	1,476 (0)	756 (0)	288 (0)
River/runoff	1,476 (0)	756 (0)	288 (0)
Ice sheet	1,800 (0)	2,772 (0)	288 (0)
Ocean	360 (1,800)	1,728 (1,044)	288 (0)
Sea ice	288 (1,476)	216 (756)	288 (0)
Ocean waves	36 (1,764)	36 (972)	288 (0)

*Note*. Model component costs estimated from the optimized processor layouts (on the Cheyenne supercomputer; https://www2.cisl.ucar.edu/resources/computational-systems/cheyenne) are presented in the bottom part of the table. Optimized processor layouts show the total number of CPUs for each model component, with the corresponding root processor in parenthesis.

**Table 2:** Comparison of Computational Cost and Wall‐Clock Time for Different Model Configurations When Running the Ice Sheet Model for ∼9,000 Years

Model configuration	Simulation cost (10^6^ core hours)	Wall‐clock time (days)
Fully coupled 1×	25.2	500
Fully coupled 10×	2.52	50
Iterative	1.68	29

*Note*. Here “Fully coupled 1×” denotes a fully coupled configuration with a synchronously ice sheets coupling, “Fully coupled 10×” is the corresponding configuration with a 10x accelerated ice sheet component, and “Iterative” denotes the iterative spin‐up procedure described in this study.

Building on the concept of asynchronous and periodic synchronous acceleration, here we present a novel iterative procedure for spinning up a coupled climate model state, targeted at computational requirements, configurations, and designs of the CESM2. This method is well suited for computationally expensive models with an interactive ice sheet component, because of uniquely long ice sheet thermal and dynamic interia. The method is designed to satisfy the following goals and requirements as well as possible:

(i) All model components are allowed to evolve and equilibrate in a coupled fashion to account for bidirectional feedback processes, including ice sheet/climate feedbacks that regulate ice sheet evolution.
(ii) The final result is an internally consistent climate state where all model components are in statistical equilibrium (or in a nonequilibrium state consistent with prior climate history, such as the Last Glacial Cycle in the case of ice sheets).
(iii) The equilibrium climate, aside from ice sheet conditions, is similar to that obtained using a standard spin‐up technique with a prescribed ice sheet geometry. Where differences arise, they can be attributed to the presence of a dynamic ice sheet.
(iv) The technique is computationally feasible for model equilibration, given available computing resources.

Our method satisfies these goals in the course of developing a feasible ice sheet/Earth system modeling and analysis workflow within CESM2. Given commonalities between CESM2 and other high‐complexity ESMs (e.g., Alexander & Easterbrook, 2015), we hope that the technique proves useful in reducing the computational cost of spinning up other coupled models, with or without interactive ice sheets.

The paper is organized as follows: The model and experiment design are presented in sections 2–4. The iterative method is demonstrated in section 5, followed by a general discussion and conclusions in sections 6 and 7.

## 2 Model Description

The CESM is a state‐of‐the‐art, global ESM, primarily developed and administered at the National Center of Atmospheric Research (NCAR). CESM2 (Danabasoglu et al., 2020) is the newest member of the CESM model family and is contributing simulations of past, present, and future climates to the CMIP6 (Eyring et al., 2016). CMIP6 is a central initiative of modern climate science and plays an important role for the upcoming IPCC Sixth Assessment Report (https://www.ipcc.ch/assessment-report/ar6/).

This study uses the publicly released version of CESM2.1.1, which consists of prognostic components of atmosphere, land, river, ocean, ocean surface waves, sea ice, and land ice; see Danabasoglu et al. (2020) and http://www.cesm.ucar.edu/models/cesm2/ for a detailed description of the model and its performance. Recent model improvements with significance for the present study are described in the following.

The Community Atmosphere Model (CAM6) has adopted the Beljaars et al. (2004) form drag parameterization and a new anisotropic orographic gravity wave scheme that accounts for the orientation of subgrid‐scale ridges and low‐level blocking. This has substantially improved precipitation on the ice sheet edges and also improved turbulent energy fluxes in the boundary layer, which are important components of the surface mass balance (SMB).

The Community Land Model (CLM5) (Lawrence et al., 2019) contains improved snow physics to account for temperature and wind‐driven compaction, and a new firn model (van Kampenhout et al., 2017) that allows for a more realistic meltwater infiltration and refreezing (the snowpack has been increased to 12 levels with a maximum depth of 10 m liquid water equivalent). The relative distribution of vegetation (bare soil + 15 different vegetation types) is prescribed in each CLM5 grid cell, but the ecosystem dynamics (i.e., life cycle and mortality) are prognostic. Also, land surface types (i.e., glaciated, vegetated, lakes, and urban) are dynamic to accommodate the transition between different surface conditions as simulated ice sheets advance and retreat in the coupled model.

The Community Ice Sheet Model (CISM2.1 Lipscomb et al., 2019) has a parallel, higher‐order velocity solver (Goldberg, 2011) that realistically simulates slow interior flow as well as fast flow in ice streams and outlet glaciers. Parameterizations of basal sliding (Aschwanden et al., 2016), iceberg calving, and other physical processes have been improved from earlier versions of the model. Surface temperature and SMB are computed in the land model (CLM5) in multiple elevation classes for each glaciated grid cell (Lipscomb et al., 2013; Sellevold et al., 2019). Over the GrIS, these fields are downscaled to the higher‐resolution CISM2 grid. The initial interpolation is bilinear in the horizontal and linear (between adjacent elevation classes) in the vertical. A global correction factor is then applied so that the total accumulation and ablation computed in CLM5 are equal to the accumulation and ablation applied in CISM2. No downscaling is currently applied for Antarctica and smaller mountain glaciers, since their geometries remain prescribed. CISM2 also includes an active isostasy model, with an elastic lithosphere and relaxing asthenosphere (with a relaxation timescale of 3,000 years) as described in Rutt et al. (2009). The bedrock topography underneath the GrIS is initialized from a relaxed state in equilibrium with the loading from the overlying ice sheet. Floating ice shelves are not modeled explicitly; instead, ice calves immediately upon floatation. We consider this a reasonable simplification for the GrIS, but it would not be appropriate for the AIS.

CESM2 has bidirectionally active ice sheet‐land‐atmosphere coupling, including an energy‐based mass‐balance scheme to represent realistic variations in accumulation and ablation, and interactive land‐surface types (including vegetated and glaciated land units). Bare ground is prescribed under the modern observed GrIS extent, while tundra is prescribed around the periphery, in regions of Greenland that are ice free at a given time. When CLM5 is run interactively with CISM2, CLM5 snow accumulation that exceeds the 10 m maximum allowed snow depth over the region that overlaps the CISM2 spatial domain (i.e., the island of Greenland) is added to the top of CLM5's snowpack, and an equivalent amount at the bottom of the snowpack is converted to ice and passed as a positive SMB to the ice sheet. Melting of the CLM5 ice column in the ice sheet domain is communicated to CISM2 as a negative SMB, with meltwater runoff (i.e., the fraction of liquid surface water that is not refrozen in the snowpack) routed from CLM5 to the ocean (Parallel Ocean Model; POP2) (Smith et al., 2010) by the river model (Model for Scale Adaptive River Transport; MOSART) (Tesfa et al., 2014). See Leguy et al. (2019) for more details on the CLM5‐CISM2 coupling.

The ice sheet model sends calving fluxes to POP2 as solid ice runoff and sends basal melting fluxes from grounded ice (usually small in comparison to surface melting and calving fluxes) as liquid runoff. In POP2, solid runoff is melted instantaneously using energy from the global ocean, and the resulting salinity and heat anomalies are spread diffusively in the surface ocean, following a normal distribution with a maximum radius of 300 km. This treatment of solid runoff also applies to the default CESM2 setup without interactive ice sheets. MOSART is currently not capable of complex subglacial hydrology, so basal meltwater is instead sent directly to the ocean model using nearest neighbor mapping. While this is not an exact emulation of more complex subglacial hydrological routing, we consider it adequate, given the small magnitude of basal fluxes and the general tendency of subglacial hydrology to route water to nearby ice sheet margins. In the simulations discussed here, because any floating ice is immediately calved, no ice shelves develop, and basal melting of floating ice shelves is thus not considered. Freshwater and salinity are conserved in the coupled model configuration. However, eustatic sea level change is not explicitly simulated by changes in the land‐ocean distribution.

By default, CISM2 is configured to run synchronously with other climate components. However, it can also be configured to run asynchronously, where the ice sheet evolves several years for each CESM2 model year. In this configuration, the ice sheet model cycles repeatedly over 1 year of SMB forcing during the accelerated period. In this case, fluxes from the ice sheet to the ocean are accumulated and averaged over the accelerated period. This methodology violates freshwater and energy conservation in the model but conversely reduces instabilities and artificial drift in the ocean model that could emerge if the accumulated freshwater flux from an accelerated ice sheet was sent to the ocean at a greater rate.

All model components are dynamically coupled and exchange state information via a coupler that conservatively interpolates fields between the different model domains. The atmosphere (CAM6) and land (CLM5) models run on a 0.9° × 1.25° finite volume grid, the ocean (POP2) model, and sea ice model (Community Sea ice Model; CICE5) use a nominal 1° resolution rotated pole grid, and the ice sheet component (CISM2) runs on a limited‐area 4 × 4 km grid centred over Greenland.

### 2.1 Component Sets

CESM2 supports a variety of model configurations and forcing protocols, ranging from standalone experiments with individual modeled Earth system components (generally forced by observational or reanalysis data sets), to coupled simulations that include all Earth system components, where the model climate is determined internally under imposed planetary boundary conditions. These model configurations, commonly referred to as component sets or *compsets*, are configured by a combined user interface/control system called the Common Infrastructure for Modeling the Earth (CIME; http://esmci.github.io/cime/). The method described here relies on the iterative use of multiple model configurations, each of which is described below.

#### 2.1.1 Fully Coupled Model (BG Component Set)

The BG component set consists of the coupled model with a two‐way interactive ice sheet, hereafter referred to as “fully coupled.” In using the term “fully coupled,” it is nonetheless recognized that further and more detailed coupling is possible—for example, in the context of the ice sheet/Earth system, bidirectional ocean‐ice sheet coupling at marine ice sheet interfaces, or via global gravitational impacts of ice sheets on sea level. This is the most comprehensive way of running CESM2 at present, where all components are interactive and the entire simulated system responds to imposed boundary conditions, such as the top of the atmosphere radiation fluxes, greenhouse gases, topography, and other planetary boundary conditions. This is also the most computationally expensive type of simulation (Table 1), though including the interactive ice sheet model only adds about 1% to the total cost of the coupled model. In the simulation described here, the fully coupled model is run with a synchronously coupled ice sheet component, meaning that the ice sheet evolves at the same rate as the CESM2 model climate.

#### 2.1.2 Coupled Model With a Data Atmosphere (JG Component Set)

As part of the methodology for this study, a new model configuration (JG component set) was introduced. This component configuration is similar to the fully coupled model configuration, but with CAM6 replaced by a computationally inexpensive data atmosphere model where the atmospheric fluxes are prescribed. We note that this configuration would be unavailable in ESMs, which, for underlying structural reasons, cannot operate without an interactively coupled atmosphere. With an optimized processor layout, throughput (i.e., simulated model years per day) is more than four times greater than for the fully coupled model configuration, and the computational cost is reduced by a factor of three (Table 1).

This model configuration allows for two‐way coupling between CISM2 and all other model components except for the prescribed data atmosphere. Most importantly, this allows GrIS meltwater runoff and calving to interact with long‐timescale ocean processes such as the Atlantic Meridional Overturning Circulation (AMOC) during convergence to steady state. Similar to standalone ocean model simulations, we apply a weak (timescale of 1 year) restoring of the sea surface salinity field to suppress spurious drift in the overturning circulation from runaway feedbacks with a prescribed atmospheric state (e.g., Griffies et al., 2009, 2016).

In the data‐atmosphere simulations described here, the ice sheet component is asynchronously coupled to the broader ESM (see section 2), in order to accelerate ice sheet spin‐up. As a result, global freshwater is not conserved during these run segments. The restoration of the surface salinity field, however, is strong enough to reduce artificial and nonphysical ocean drift, which furthermore approaches zero as residual drift in ice sheet volume decreases.

#### 2.1.3 Ice Sheet Only (T Component Set)

The ice sheet component (CISM2.1) can be run as a free‐standing (i.e., ice sheet only) model within the CIME infrastructure using the T component set. In this configuration, SMB and surface temperature on multiple elevation classes in each land grid cell are downscaled to CISM2 to account for elevation feedbacks as the ice sheet evolves. Typically, the surface forcing is provided by a previous run with an active land model. The computational cost of the ice sheet‐only configuration is low compared to the coupled model configurations described above, allowing for multimillennial simulations within a wall‐clock day (Table 1). The ice sheet‐only simulations described here were driven with prescribed boundary conditions from the fully coupled simulation segments of the spin‐up procedure.

### 2.2 Dynamic Topography Updating

In the standard implementation of CESM2, the topography used by the atmosphere model (CAM6) is time invariant. In standard model configurations without interactive ice sheets, this is acceptable. However, it introduces an inconsistency when running with interactive CISM2—particularly multicentennial or multimillennial simulations—as ice sheet topography changes over time. Updating the CAM6 topography continually at run time is currently not practical, as information about subgridscale topography variance and ridge orientation (used in orographic drag and gravity wave parameterizations in the planetary boundary layer) are derived from a high‐resolution global data set using algorithms that are not included or optimized for runtime CAM6 operation.

Thus, as part of the experiments presented here, an off‐line tool was developed that periodically updates the topography boundary condition in CAM6, to include changes in the ice sheet topography. Since this topography updating routine is not officially supported in the CESM2 model distribution, we briefly outline the workflow in the following:

(i) At the completion of each run segment, atmosphere and ice sheet (GrIS) states are written to standard restart files.
(ii) The ice sheet topography is extracted from the CISM2 restart file, regridded to a 30‐s grid (approximately 1‐km resolution) and overlaid onto the GMTED2010 data set (Global Multi‐resolution Terrain Elevation Data 2010; Danielson & Gesch, 2011), which forms the basis for the CAM6 topography.
(iii) The CAM6 topography generation routine (Lauritzen et al., 2015) is then run in its entirety. This includes remapping of the modified GMTED2010 topography to a 3‐km cubed sphere grid, from which the subgridscale topography variance and ridge orientation are derived. The topography is then smoothed and interpolated back to the CAM6 model resolution.
(iv) The new global, smoothed CAM6 topography and subgrid roughness fields, which incorporate the altered CISM2 topography, are reinserted into the CAM6 restart and topography files.
(v) CESM2 is then automatically resubmitted.

## 3 Model Spin‐up Procedure

The primary objective of spinning up (or equilibrating) an ESM is to generate an internally consistent coupled Earth system state, where all model components are in a statistical equilibrium (or in a nonequilibrium state consistent with prior climate history, in the case of ice sheets). This state reflects an internal balance among all intercomponent fluxes, states, interactions, and feedbacks. The length of a model spin‐up is practically determined by the longest equilibration time of included Earth system components. In the absence of ice sheets, ESM equilibration times are typically determined by abyssal ocean and deep soil conditions, which can carry traces of past climate in dynamic, thermodynamic, and geochemical conditions for several millennia. Inclusion of an interactive ice sheet component complicates the exercise further, since ice sheets can carry a dynamic and thermodynamic memory spanning well over 10 kyr. This makes ice sheets the Earth system component with the longest spin‐up timescale in coupled ESMs, by a wide margin.

In contrast to ice sheets, the atmosphere has almost no memory and adjusts quickly to altered conditions. At the same time, atmospheric models are often the most computationally expensive parts of coupled models. In CESM2, for example, the atmosphere component (CAM6) accounts for about 70% of the total coupled model cost at the standard 1° resolution (based on the optimized layout in Table 1). Thus, substantial computational savings can be made by minimizing atmosphere model integration, while still allowing all model components to evolve in a manner that mimics a fully coupled simulation. In particular, we assume that if the model climate is in quasi‐equilibrium (i.e., long‐term climate drift is small compared to interannual variability), the atmospheric component can be prescribed for extended periods of time without significantly affecting the spin‐up trajectory of components with higher inertia or equilibration timescales. However, to regain consistency with a coupled simulation, the atmospheric state has to be updated periodically via fully coupled simulations, to allow atmospherically regulated changes in the overall coupled model state to evolve realistically.

**Figure 1:**
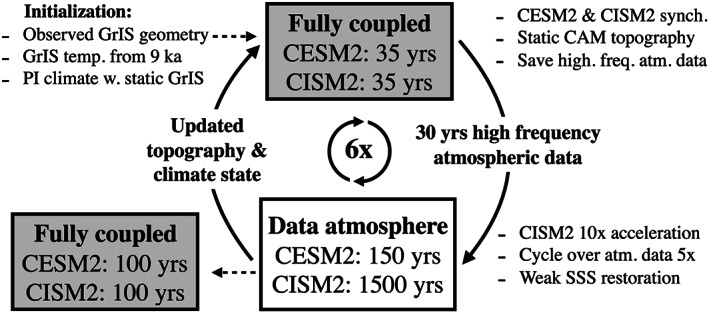
Schematics illustration of the iterative spin‐up procedure. The fully coupled simulations were run for 35 years with a synchronous climate‐ice sheet coupling. High‐frequency atmospheric fluxes were extracted from the last 30 years. Subsequently, the coupled model with a data atmosphere was run for 150 years with a 10 times acceleration of the ice sheet component (1,500 ice sheet years), looping five times over the atmospheric data archive. The CAM topography was updated before a new fully coupled simulation was run, thus starting the cycle over. A total of six iterations were run, followed by a 100 yearlong fully coupled simulation to remove inconsistencies from the iterative procedure. This resulted in 9,310 CISM2 years and 1,210 simulated years with the ocean, sea ice, and land models. SSS denotes sea surface salinity.

The workflow of the spin‐up procedure is as follows (see also schematic illustration in Figure 1):

(i) A fully coupled simulation (section 2.1.1) is run for 35 model years, using full synchronous climate‐ice sheet coupling. We only consider data from the last 30 model years (the first 5 years of the output data are discarded for the first and all subsequent coupled simulation iterations) to remove any initial spurious transient behavior in the atmospheric state resulting from the iterative procedure. During this stage, instantaneous high‐frequency atmospheric data are extracted from the coupler and written to external files. We chose 30 years as a compromise between simulation cost and accuracy and to ensure that several cycles of naturally occurring modes of variability on subseasonal to subdecadal timescales—e.g., ENSO and NAO—are represented in this high‐frequency data archive. Data consist of hourly longwave and shortwave surface radiation and near‐surface horizontal (*u* and *v*) wind, as well as 3‐hourly surface temperature, pressure, precipitation, and near‐surface (i.e., evaluated at the lowest model level) potential temperature, specific humidity, density, and elevation.
(ii) Next, the simulation continues using the model configuration where the interactive, coupled atmosphere model is replaced by the high‐frequency data archive (section 2.1.2) generated from the preceding coupled simulation. The 30 years of atmosphere data are cycled five times, for a total integration length of 150 model years for the ocean, land, and sea ice components. Concurrently, the ice sheet component is accelerated by a factor of 10, for a total of 1,500 simulated ice sheet years. The ocean surface salinity relaxation (described in section 2.1.2) uses climatological data from the preceding coupled simulation. At the end of the simulation segment, the topography data set used by the atmospheric model in the coupled model configuration is updated (see section 2.2) to incorporate changes in the GrIS topography and spatial extent.
(iii) Steps (i) and (ii) are repeated for as many iterations as required.
(iv) As a final step, the coupled model is run for 100 years with synchronous climate‐ice sheet coupling to remove any residual inconsistencies resulting from the iterative procedure and provide a basis for assessing internal consistency and equilibration.

In the spin‐up documented in this study, we applied this procedure for a total of six fully coupled/data‐atmosphere‐model iterations. After six iterations and the final fully coupled simulation, the ice sheet model has run for a total of 9,310 years (=6 × 35 + 6 × 1,500 + 100), while the ocean, land, and sea ice components have each run for 1,210 years (=6 × 35 + 6 × 150 + 100). The atmosphere has run for 310 years (=6 × 35 + 100)—a factor of ∼4 less than the ocean, land, and sea ice and a factor of ∼30 less than the ice sheet. As a result, the total computational cost (see Tables 1 and 2) is approximately equal to that of a 600‐year simulation with the coupled model (1.68 M core hours/2.8 k core hours/year ≈ 600 years). In section 5 we validate the ability of this less‐expensive spin‐up workflow to adequately replicate coupled climate conditions generated by a standard CESM2 simulation with a prescribed GrIS geometry. This comparison provides a key benchmark of success for the overall procedure.

## 4 Initial and Boundary Conditions

### 4.1 Boundary Conditions

The iterative procedure described in section 3 was carried out under a constant preindustrial model forcing protocol, consisting of observed land‐ocean distribution, and 1850 CE greenhouse gas concentrations, orbital parameters, vegetation, and land use.

The initial (*t* = 0) topographic boundary condition in the atmosphere model is prescribed to modern observations and then updated as part of the iterative procedure to include changes in the GrIS topography as the ice sheet evolves (see section 3).

### 4.2 Initial Conditions: Climate

All *non‐ice sheet* model components were initialized from a multicentennial, coupled, preindustrial simulation with a development version of CESM2. In this prior simulation the GrIS was prescribed at its observed area and topography (Morlighem et al., 2014).

This simulation also serves as initial condition for the 1,200‐year preindustrial control simulation for CMIP6 (Danabasoglu et al., 2020) (from here referred to as piControl) with prescribed GrIS geometry. We use this noninteractive ice sheet simulation as a benchmark for assessing viability and overall success of the iterative procedure (section 5), in terms of similarity between final non‐ice sheet component states between the two cases. To facilitate this comparison, both the piControl and the present simulation adopted an identical preindustrial forcing protocol following the CMIP6 guidelines (Eyring et al., 2016).

### 4.3 Initial Conditions: Ice Sheet

The ice sheet component was initialized using a protocol broadly similar to that of the CISM2 contribution to initMIP‐Greenland (a model intercomparison project under CMIP6, focusing specifically on ice sheet model initialisation; Goelzer et al., 2018). The ice sheet thickness and extent were initialized from the modern observed GrIS geometry and bedrock elevation (Morlighem et al., 2014), but with peripheral glaciers and ice caps removed. These were removed for reasons related to calculation of the SMB on multiple elevation classes on the nominal 1° land‐model grid (Sellevold et al., 2019). This method works well for large ice sheets that are explicitly resolved on the coarse land‐model grid but becomes less appropriate for smaller glaciers where seasonal ablation zones are poorly represented. Thus, glaciers and ice caps were removed from the initial conditions. Furthermore, the model was modified to suppress glacial inception outside of the contiguous ice sheet. These modifications are included as a namelist option in CESM2.1.1. This was found to be necessary to prevent runaway feedback processes resulting in near‐total glaciation of the Greenland continent. They do not, however, inhibit the ice sheet from expanding outside its initial footprint due to overall mass balance and flow dynamics.

GrIS internal temperature was initialized with a temperature structure corresponding to the 9 ka GrIS state (9,000 years before present), as developed in the long ice sheet simulation of Fyke et al. (2014), after interpolation along the vertical sigma coordinates to the observed GrIS geometry. The simulation in Fyke et al. (2014) was run over a full glacial cycle (from 130 ka to modern), using temporal variations in Greenland *δ*^18^O (the ratio of heavy and light oxygen isotopes derived from Greenland ice cores) as a proxy for climate evolution. Thus, initializing the 9 ka thermal state from this prior standalone ice sheet simulation represents the estimated residual thermal memory of the last glacial period, which in turn influences the final spun‐up preindustrial ice rheology at the end of the ∼9,000‐year spin‐up procedure.

Given a constant preindustrial forcing protocol, the time needed to spin up the GrIS is expected to be roughly equal to the average ice sheet residence time—that is, the time an average ice parcel spends in the ice sheet before returning to the ocean or atmosphere via calving, runoff, or sublimation. Given the modern GrIS volume of 2.93 × 10^15^ m^3^, with a total ice sheet mass above flotation of roughly 2.69 × 10^18^ kg (Morlighem et al., 2017) and an annually integrated GrIS SMB of ∼350 × 10^12^ kg/year (Noël et al., 2018), the average GrIS residence time is around (total mass/integrated SMB) ∼7,700 years. While not meant as a statement on actual parcel‐specific residence times, which can range from months to millennia, this heuristic supports our use of a spin‐up length of ∼9,000 model years (and consequent choice of a 9 ka initial temperature structure), which is largely consistent with previous spin‐up exercises with standalone ice sheet models (e.g., Koenig et al., 2015; Stone et al., 2010).

## 5 Spin‐up Demonstration

To demonstrate the applicability and viability of the spin‐up procedure, we now demonstrate that (i) the climate simulated by the procedure behaves predictably throughout the iterative spin‐up, including transitions between the coupled and data atmosphere simulation segments; (ii) the model climate converges toward an equilibrium state sufficiently similar to the piControl simulation with a prescribed GrIS geometry, excepting physically realistic differences resulting from the presence of a coupled ice sheet; (iii) the final ice sheet state is consistent with prior climate history; and (iv) no significant biases in the final simulated GrIS state result from the spin‐up procedure itself, as opposed to biases inherent to the CESM2 climate or CISM2 representation of ice sheet physics. We explore each of these findings in the following sections.

### 5.1 Climate: Large Scale Evolution of Oceanic Conditions

**Figure 2:**
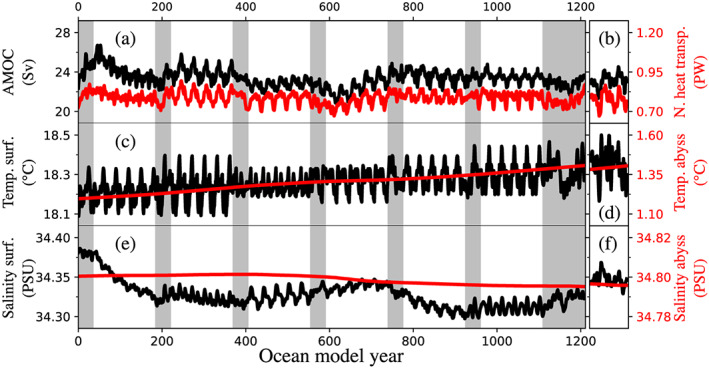
Annual mean time series comparing the iterative spin‐up procedure (left) with 100 years of the piControl simulation with prescribed GrIS geometry (right). (a, b) Atlantic meridional overturning circulation (AMOC; maximum meridional overturning streamfunction below 500 m and north of 28°N; black) and northward heat transport at 50°N (red); (c, d) global mean sea surface temperature (black) and abyssal ocean (red); (e, f) global mean sea surface salinity (black) and salinity in abyssal ocean (red). Fully coupled segments are indicated by gray background in panels (a), (c), and (e); The coupled model with a data atmosphere was used elsewhere.

Large‐scale oceanic conditions are sensitive to spin‐up methodology (section 2.1.2). Thus, similarity of large‐scale ocean conditions generated by the methodology presented here to that developed by standard climate model spin‐ups is an important test of the overall approach. In support of this comparison, Figure 2 compares the time evolution of ocean diagnostics from the iterative procedure to the last 100 years of piControl. By construction, the interannual variability in the fully coupled segments (gray background shading in left panels in Figure 2) is repeated in the simulation segment with a data atmosphere that follows immediately after (white background shading in left panels in Figure 2). This is particularly notable in AMOC strength and surface ocean conditions, which both exhibit a pronounced year‐to‐year variability. In contrast, fields in the abyssal ocean are decoupled from atmospheric conditions on annual timescales and therefore do not generally exhibit high‐frequency variability.

The top panel in Figure 2 shows AMOC evolution. Overall, AMOC strength is relatively stable over the course of the simulation, though it exhibits episodes of both increased and decreased activity. For example, the overturning becomes almost 10% more vigorous in the first fully coupled segment after model initialization, presumably because of a locally disrupted hydrological cycle in the North Atlantic due to rapid changes in the GrIS geometry (see section 5.3). This intensification, however, is curbed in the first simulation segment with the data atmosphere, and the AMOC strength returns to a similar level as in the initial condition after ∼200 simulated ocean years. Subsequently, AMOC strength remains fairly stable, with the exception of a slight weakening after about 600 ocean model years and in the early stages of the extended fully coupled segment at the end of the model integration (rightmost period indicated by gray background shading in the top left panel). Final AMOC strength at the end of the spin‐up procedure is comparable to the traditional spin‐up simulation with prescribed GrIS geometry (cf. Figure 2b).

The meridional heat transport (evaluated at 50°N; red contour line in Figure 2a) is closely related to the overturning circulation and therefore exhibits a broadly similar temporal variability as the AMOC strength. The meridional heat transport at the end of the simulation is in close agreement with that from the traditional spin‐up.

Similar to the AMOC and associated meridional heat fluxes, global average ocean temperature is comparatively stable (Figure 2c), though there is a small but systematic warming trend over the course of the simulation. The residual temperature trend in the abyssal ocean is about 0.02°C per century (approximately 0.25°C in 1,210 years). This trend is consistent with piControl spin‐up simulation (cf. Figures 2c and 2d), indicating that the iterative procedure described here is likely not responsible.

Finally, the lower panels (Figures 2e and 2f) show the temporal evolution of global ocean salinity. Abyssal ocean salinity (red line) exhibits almost no drift over the course of the simulation. The surface salinity, on the other hand, decreases by about 0.05 PSU in the first 200 years but then remains relatively stable.

These comparisons collectively indicate that the spin‐up procedure does not introduce unexpected or spurious signals into large‐scale simulated oceanographic circulations, despite significant reductions in computational cost.

### 5.2 Climate: Equilibrium State

**Figure 3:**
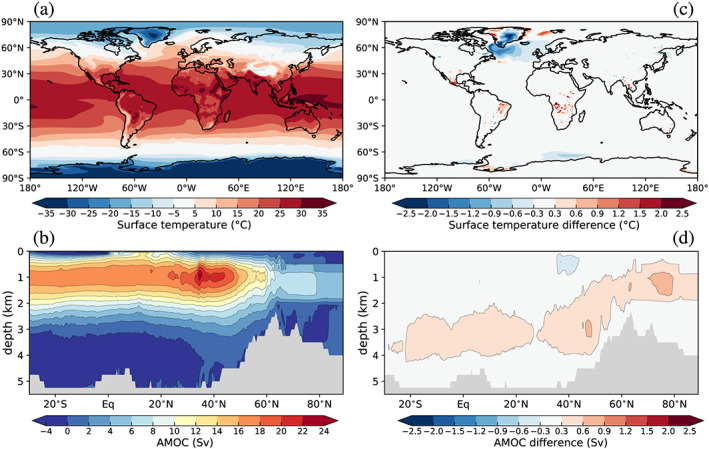
The 100‐year annual climatologies of (a) surface temperature and (b) Atlantic meridional overturning circulation from the end of the iterative spin‐up simulation. Panels (c) and (d) show equivalent differences with respect to a 100‐year climatology from the piControl simulation with a prescribed GrIS geometry. Gray shading in panels (b) and (d) indicate bathymetry; differences that are not statistically significant at the 99% level (using a *t* test) are masked out.

**Figure 4:**
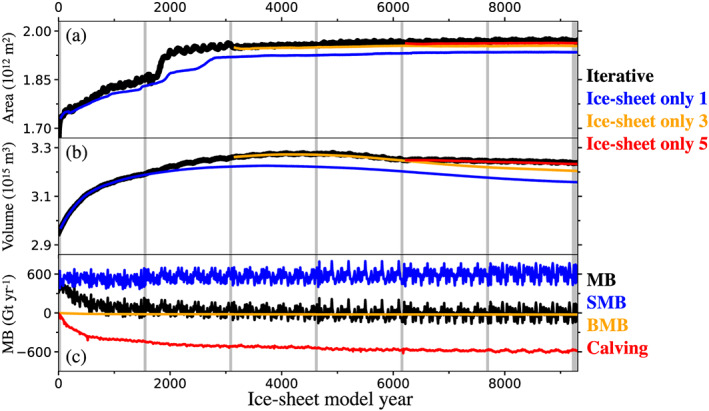
Time evolution of GrIS: (a) area, (b) volume, and (c) mass balance components (net bass balance MB, black; surface mass balance SMB, blue; basal mass balance BMB, orange; calving, red). Black lines in panels (a) and (b) indicate the iterative spin‐up simulation; colored lines indicate ice sheet‐only simulations. Fully coupled segments are indicated by gray vertical background bars; the data atmosphere model configuration was used elsewhere. Note that the time axis is extended relative to Figure 2 due to (10 times) ice sheet acceleration in data atmosphere segments.

The final state achieved via the new spin‐up methodology should be similar to that achieved from the standard spin‐up technique. Figure 3 compares 100‐year annual climatologies derived from the end of the iterative simulation with the end of the piControl spin‐up simulation with prescribed GrIS geometry. Both simulations converge toward the same overall climate state, with the exception of an appreciable cold anomaly over Greenland and the northern North Atlantic in the iterative simulation. Cool temperatures over the ice sheet result primarily from elevation differences between the two simulations (elevation differences of >100 m are simulated over the central parts of the ice sheet; see section 5.4). Subsequently, cold air developed over the GrIS interior is likely advected over the surrounding ocean by mean simulated atmospheric flow, causing off‐ice sheet cold anomalies. Also, the coupled GrIS interacts directly with the ocean through runoff fluxes and solid ice discharge (calving) in marine‐terminating outlet glaciers, which adds relatively more ice during summer months to regional seas than in piControl. This in turn decreases summertime temperatures, resulting in further regional cooling. As discussed further in sections 5.3 and 5.4, the ice sheet at the end of the iterative procedure is larger than modern observations, which results in overall increased freshwater fluxes relative to piControl (the average SMB has increased by around 100 Gt/year compared to the initial state; see Figure 4 and Table 3). Cooler sea surface temperatures resulting from GrIS coupling likely amplify density‐driven deep convection in the North Atlantic, leading to a minor strengthening of the North Atlantic deep water branch of the meridional overturning circulation (Figures 3b and 3d), relative to the piControl.

**Table 3:** Average and Standard Deviation (in Brackets) of Ice Area, Ice Volume, Total Mass Balance, Surface Mass Balance, Calving Flux (Solid Ice Discharge), and Basal Mass Balance From Each Run Segment in the Iterative Procedure

	FC1	DA2	FC2	DA3	FC3	DA4	FC4	DA5	FC5	DA6	FC6	DA7	FC7
Ice sheet year	35	1,535	1,570	3,070	3,105	4,605	4,640	6,140	6,175	7,675	7,710	9,210	9,310
Ice area	1.705	1.804	1.853	1.929	1.950	1.955	1.956	1.964	1.963	1.968	1.966	1.971	1.968
(10^12^ m^2^)	[0.022]	[0.034]	[0.003]	[0.019]	[0.001]	[0.003]	[0.001]	[0.003]	[0.001]	[0.002]	[0.001]	[0.002]	[0.001]
Ice volume	2.958	3.124	3.192	3.234	3.261	3.272	3.276	3.266	3.248	3.249	3.243	3.242	3.235
(10^15^m^3^)	[0.005]	[0.059]	[0.001]	[0.019]	[0.000]	[0.004]	[0.000]	[0.009]	[0.000]	[0.002]	[0.000]	[0.002]	[0.001]
Total mass balance	457	139	78	44	34	13	−4	−13	10	1	9	0	−9
(Gt/year)	[70]	[123]	[72]	[70]	[68]	[66]	[81]	[78]	[69]	[70]	[84]	[83]	[83]
Surface mass balance	490	510	540	557	568	560	560	573	593	601	603	611	591
(Gt/year)	[65]	[70]	[72]	[70]	[69]	[69]	[81]	[78]	[70]	[74]	[85]	[86]	[83]
Calving flux	−31	−356	−443	−493	−513	−526	−542	−563	−560	−577	−571	−587	−576
(Gt/year)	[17]	[92]	[5]	[20]	[2]	[11]	[3]	[11]	[2]	[9]	[3]	[13]	[4]
Basal mass balance	−2	−15	−19	−20	−21	−21	−22	−23	−23	−23	−23	−24	−24
(Gt/year)	[0.167]	[4.618]	[0.096]	[0.326]	[0.0394]	[0.498]	[0.033]	[0.341]	[0.0282]	[0.151]	[0.056]	[0.299]	[0.076]

*Note*. Positive values represent ice mass gain. The total mass balance is the sum of mass accumulation from surface mass balance, and mass loss from calving fluxes and basal mass balance. Standard deviations are based on area averaged quantities, using the full time series of each run segment. The abbreviations FC and DA denote the fully coupled and data atmosphere model configurations.

Differences between the iterative spin‐up and piControl (Figure 3c) also reveal warm anomalies scattered across tropical land areas. The origin of these warm anomalies is unclear, but, owing to their patchy appearance, we speculate that they are due to local differences in vegetation cover between the two simulations, related life cycle processes that are functions of local time‐evolving temperature, precipitation, and soil nutrients. Differences in these factors between the two simulations may relate to small differences in the location and intensity of precipitation in the intertropical convergence zone, which results in slight seasonal variations in gross primary production (not shown). This in turn may be at least partially explained by the North Atlantic cold anomaly in the final state of the iterative spin‐up simulation, suggesting that tropical land area differences are not a spurious artifact of our methodological design.

Taken together, the comparisons presented in this and the previous section support a conclusion that the iterative spin‐up approach is not unduly impacting broader (non‐ice sheet) conditions, as represented by piControl.

### 5.3 Ice Sheet: Temporal Evolution

**Figure 5:**
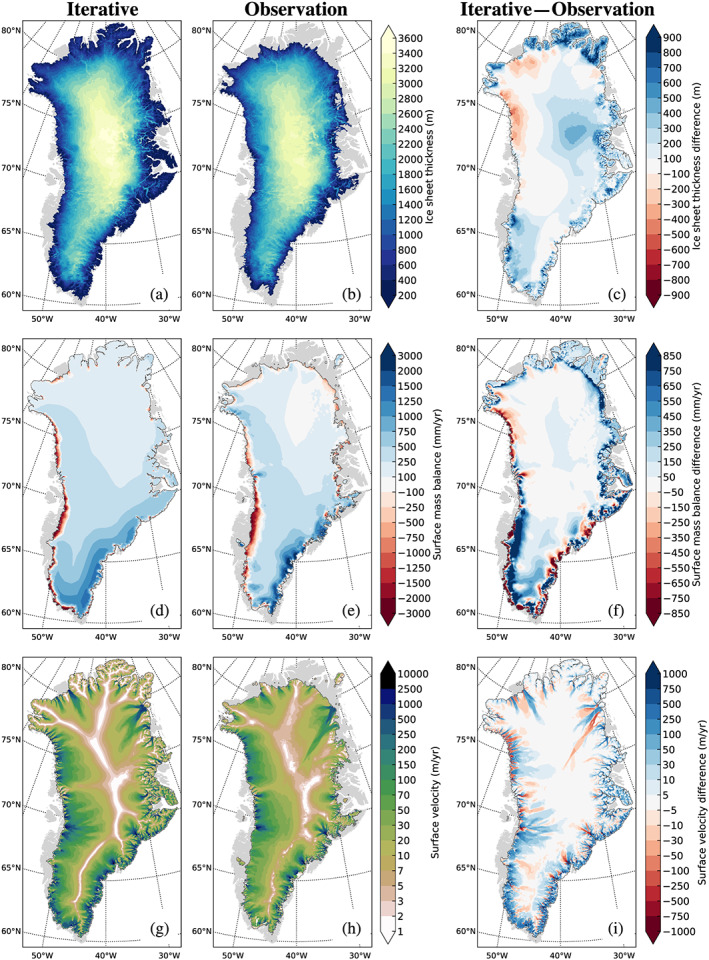
(a) Simulated GrIS thickness, (b) observed GrIS thickness (Morlighem et al., 2014), and (c) difference between simulated and observed thickness. (d) Simulated surface mass balance, (e) average RACMO2.3 1960–1989 surface mass balance (Noël et al., 2015), and (f) difference between simulation and RACMO2.3. (g) Simulated ice sheet surface velocity, (g) observed ice sheet velocity (Joughin et al., 2010), and (i) difference between simulated and observed velocities. All fields in the left column are evaluated as 50‐year averages from the end of spin‐up simulation. Gray shading indicates ice‐free continent.

GrIS geometry and mass balance evolution during the course of the iterative spin‐up are shown in Figure 4. CESM2 qualitatively reproduces the spatial pattern of climatological observed GrIS SMB well, as evidenced by Figure 5 and in following discussions. However, net positive precipitation biases in the ice sheet interior and over peripheral tundra render the equilibrium GrIS substantially larger than modern observations, as ice mass accumulates during the multimillennial spin‐up simulation (Table 3). We note, however, that these biases are a common feature of CESM2 simulations of the GrIS and do not arise from the iterative spin‐up approach itself.

Figure 4 and Table 3 highlight that over the course of the iterative spin‐up simulations, the ice sheet grows somewhat piecemeal, with rapid bursts just after initialization and after about 2,000 model years, followed by more quiescent periods with smaller changes in ice sheet geometry. The largest single area expansion occurs around model year 2,000, as the ice sheet dynamically expands into the tundra in the far north (see Figure 5). However, these regions have a low annual precipitation, and the new ice is thin. The large area expansion is thus not reflected in a corresponding increase in the overall ice sheet volume (Figure 4b and Table 3).

Ice sheet area also increases rapidly in the first decades of the simulation (Table 3). This expansion is partly explained by a mass imbalance prompted by the CISM2 initial conditions. Both the calving flux and basal mass balance are initially small, while the SMB, which is calculated in the land model, is already well developed (see Figure 4c and Table 3). Initial SMB is 490 ± 65 Gt/year, considerably higher than the modern (1960–1990 CE) estimate of ∼350 Gt/year (Noël et al., 2018). SMB subsequently increases and stabilizes at ∼600 Gt/year after approximately 5,000 model years (Table 3). This increase is largely a function of positive feedbacks between ice sheet area and SMB, because as the area grows, the accumulation area and accumulation/ablation area ratio grow, promoting further growth.

The initial mass imbalance corresponds to a global mean sea level decrease of about 1.3 mm/year. In comparison, the residual drift at the end of the simulation (end of the last fully coupled segment) is 0.03 mm/year, with a standard deviation of ±0.23 mm/year. Interannual variability in total mass balance is dominated by SMB variability (Table 3), while the other components—calving flux and basal mass balance—account for much less variability. The total mass balance at the end of the simulation is −9 ± 83 Gt/year, with contributions of 591 ± 83 Gt/year from SMB, −24 ± 0.08 Gt/year from basal mass balance, and 577 ± 4 Gt/year from calving (see Table 3).

### 5.4 Ice Sheet: Spatial Fields

The final GrIS area is ∼15% larger than modern observations (Table 3), with the difference coming from glaciation of peripheral tundra that is ice free in reality. In this final state, ice reaches the sea in many regions, with the notable exception of a number of smaller areas in the far north, and much of the southwestern margin, which, in broad agreement with observations, remains far inland (Figures 5a–5c). Consistent with a positive bias in ice area, ice volume is also overestimated, with large thickness anomalies in the north‐central interior and the southwestern parts of the ice sheet. These elevation differences are related to positive precipitation anomalies over the majority of the ice sheet interior. Conversely, anomalously low precipitation leaves the ice thickness underestimated along the northern and northwestern margins. Final ice volume is approximately 8.3 m sea level equivalent, which exceeds observation estimates of 7.4 m sea level equivalent (Morlighem et al., 2017) by around 12%. While this bias is significant, we emphasize that (i) it is consistent in sign with biases in many other GrIS simulations using a spin‐up (rather than data assimilation) approach and (ii) the bias is not a result of the spin‐up procedure itself but rather reflects intrinsic climate and ice sheet model behavior in CESM2.

In broad agreement with previous studies (e.g., van Kampenhout et al., 2019), CESM2 captures the overall spatial SMB patterns but struggles to represent local spatial gradients. In particular, precipitation is generally underestimated on the ice sheet periphery and overestimated in the interior. This is at least partly related to the coarse grid resolution in the atmosphere model, which is unable to capture steep ice sheet margins and thus underestimates orographic precipitation in coastal areas and along the ice sheet edges (van Kampenhout et al., 2019). The extent and magnitude of melt‐driven ice loss simulated by CESM2 compares well to the actual melt experienced in ablation areas along the western GrIS margin. Positive SMB biases are present in the far north and along the eastern margin where the ice sheet has expanded well outside of its modern observed footprint. These regions are tundra in the current climate, and the SMB is thus effectively zero here for the purpose of SMB bias assessment.

Despite SMB biases, the simulated ice sheet velocity distribution is broadly similar to observations (Joughin et al., 2010). Simulated outlet glaciers are more numerous than in reality because of a larger fraction of the ice margin that is marine terminating. The highest ice stream velocities tend to be underestimated (Figure 5); for example, the highest simulated surface velocity is 6 km/year, which is substantially lower than the 10 km/year that is regularly observed in Jakobshavn Isbræ (e.g., Joughin et al., 2004; Rignot & Mouginot, 2012). This comparison, however, is somewhat biased, as the higher observed velocities in Jakobshavn Isbræ are measured in a comparatively warmer climate without a buttressing ice tongue. Moreover, northwest quadrant ice streams are narrower and extend farther inland than in observations. In contrast, the simulated Northeast Greenland Ice Stream is more diffuse and does not extend as far inland as observed, consistent with standalone CISM2.1 (Lipscomb et al., 2019). The model also simulates internal multimillennial oscillations of the Northeast Greenland Ice Stream and the Humboldt glacier, resulting in horizontal ice stream migrations, which may help explain regional differences from observations that (to the extent this oscillation is realistic) likely only captures one phase of the long‐term variability.

**Figure 6:**
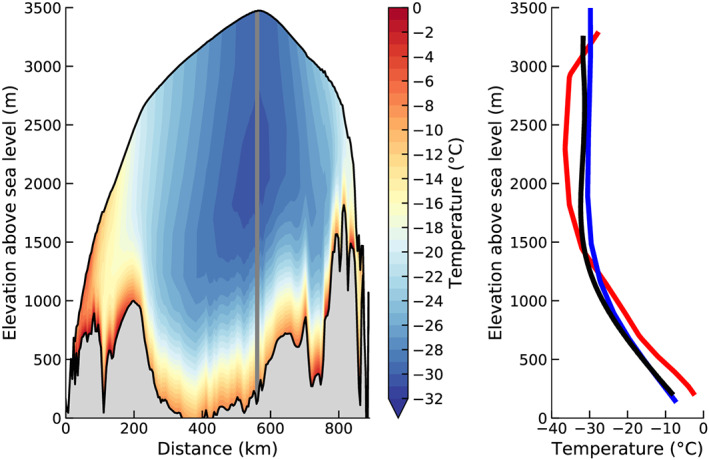
(a) Cross section of temperature across the central ice sheet at the end of the spin‐up simulation. (b) Comparison of vertical temperature profiles at summit location (72.6°N, 37.6°W; indicated by gray vertical bar in the left panel) to the GRIP temperature profile; red: temperature from initial conditions (9 ka profile in Fyke et al., 2014); blue: temperature at the end of spin‐up procedure. Note that the residual subsurface cold anomaly is not clearly visible because of the range on the horizontal axis. Black: GRIP temperature profile (Dahl‐Jensen et al., 1998).

The ice sheet's internal temperature structure plays a role in regulating ice rheology and internal flow dynamics. Figure 6 plots a vertical temperature cross section through the summit location of the equilibrium ice sheet. The temperature profile at Summit closely matches the in situ temperature profile from the GRIP core (Dahl‐Jensen et al., 1998) (cf. blue and black lines in Figure 6b), highlighting similarities in summit surface air temperatures between CESM2 and observations. Notably, although most of the thermal memory of the initial condition has vanished after 9,000 model years (cf. red and blue lines in Figure 6b), the internal simulated GrIS temperature profile still retains a weak thermal signature of the Last Glacial Period, manifested as a subsurface cold anomaly in the interior of the ice sheet (Figure 6a), which reflects recent emergence from the Last Glacial Period; see further discussion in Fyke et al. (2014). This internal temperatures assessment indicates that the iterative spin‐up approach successfully retains the internal ice thermal and viscoelastic memory of the Last Glacial Period.

#### 5.4.1 Ice Sheet: Comparison With Ice Sheet‐Only Simulations

The simulations discussed thus far include time‐varying, two‐way interactive climate‐ice sheet coupling. To provide an assessment of the effect of coupling on ice sheet spin‐up characteristics, we conducted a parallel set of ice sheet‐only simulations (section 2.1.3) with a time‐invariant SMB forcing (Lipscomb et al., 2013; Sellevold et al., 2019). These experiments were branched from the fully coupled segments of the iterative procedure at various points along the spin‐up trajectory (using 30 consecutive years of SMB forcing from each branch point) and were run for lengths corresponding to the end of the main spin‐up simulation. Elevation feedbacks are implicitly accounted for when downscaling the SMB forcing to the CISM2 grid in these ice sheet‐only simulations. Nevertheless, comparisons with the iterative simulation provides a qualitative assessment of how the spun‐up ice sheet state is influenced by interactive climate‐ice sheet coupling.

Results from this comparison are shown in Figure 4 and Table 4. The GrIS geometry evolves in a broadly similar fashion in both experiments (cf. colored lines in Figures 4a and 4b). However, the ice sheet‐only simulations consistently simulate a smaller GrIS area and volume than the main experiment, indicating that interactive climate‐ice sheet coupling favors growth (Figures 4a and 4b and Table 4). Since the general climate forcing itself favors growth, this suggests a net sum of Earth system‐ice sheet feedbacks that is positive, as represented in the simulation.

**Table 4:** Volume and Area of Ice Sheet Model Simulations (Section 2.1.3) and the Equivalent Ice Sheet From Year 9310 From the Iterative Procedure

	Length	Volume	Area
Simulation	(Years)	(10^15^ m^3^)	(10^12^ m^2^)
Ice sheet only 1	9,310	3.16	1.93
Ice sheet only 2	7,775	3.18	1.96
Ice sheet only 3	6,240	3.20	1.95
Ice sheet only 4	4,705	3.20	1.96
Ice sheet only 5	3,170	3.23	1.96
Ice sheet only 6	1,635	3.23	1.96
Ice sheet only 7	100	3.23	1.96
Iterative	9,310	3.24	1.97

*Note*. The standalone ice sheet model simulations using 30 years of SMB forcing were branched from fully coupled segments in the iterative simulation and run until the nominal ice sheet year matched the end year of the iterative simulation. Here “ice sheet only 1” denotes the first ice sheet model simulation that was branched from the first fully coupled segment of the iterative procedure and was run for 9,310 years, and so forth.

**Figure 7:**
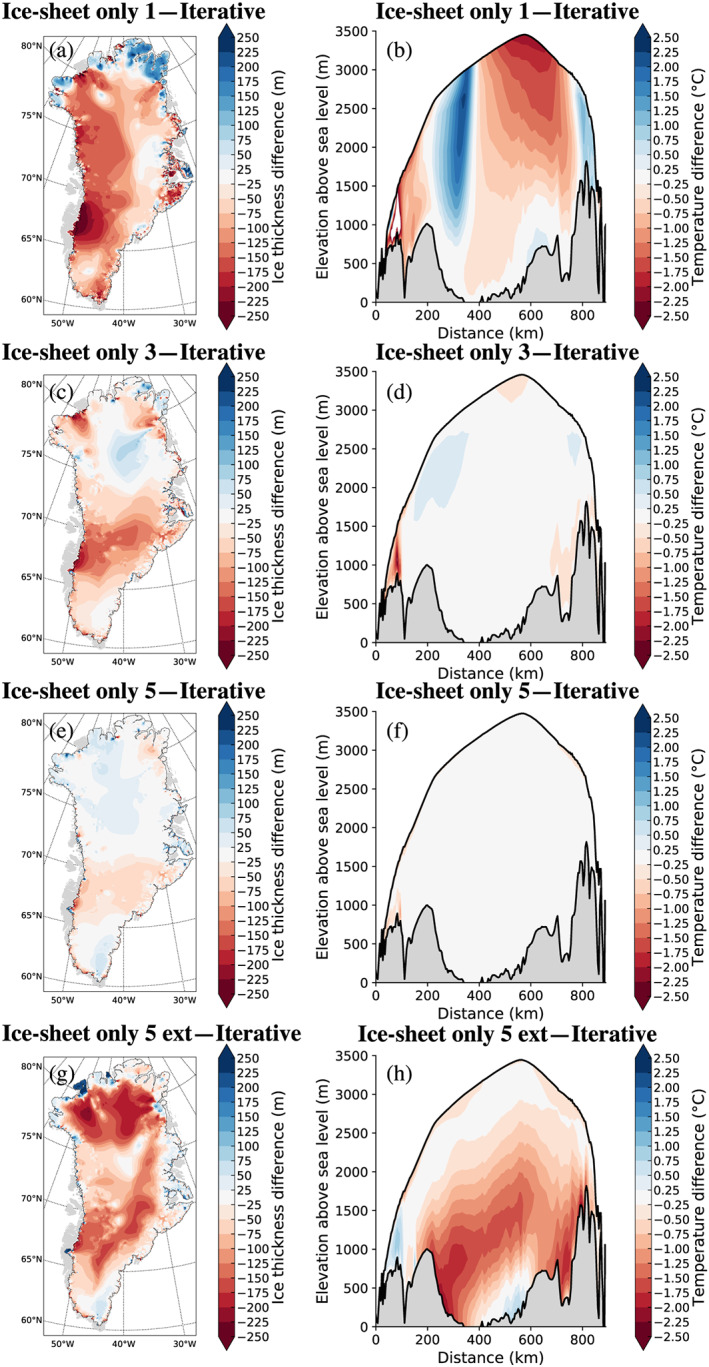
Difference in ice thickness (left column) and vertical temperature cross section (right column) between standalone GrIS model simulations and the GrIS resulting from the iterative procedure in (a, b) first ice sheet only simulation (branched from ice sheet year 0); (c, d) third ice sheet only simulation (branched from ice sheet year 3,070); (e, f) fifth ice sheet only simulation (branched from ice sheet year 6175); and (g, h) fifth ice sheet only simulation extended to 10,000 years beyond the end of the iterative procedure.

The importance of two‐way interactive climate‐ice sheet coupling is even more apparent in Figure 7, which compares the thickness and vertical temperature between the ice sheet from the end of the iterative spin‐up procedure, and the ice sheet‐only simulations. The first ice sheet‐only simulation, using SMB forcing from the first fully coupled simulation, converges toward a state with substantial elevation differences (of order 100 m) over almost the entire domain. Additionally, the internal temperature distribution is generally biased warm relative to the same distribution from the iterative spin‐up procedure, consistent with a surface temperature forcing that is based on lower ice sheet topography than that developed by the iterative procedure.

Differences between the standalone and coupled simulations become progressively smaller for ice sheet‐only simulations branched from the main experiment at later points, and simulations started after year 5,000 are almost indistinguishable from the main experiment. However, we emphasize that the spun‐up GrIS state at the end of the iterative procedure is not an equilibrium state. Rather, it reflects a state that is consistent with the preindustrial model climate, while simultaneously retaining a small residual drift that is compatible with the climate history as far back as the last glacial period.

The ice sheet thickness and internal temperature continue to evolve when running the ice sheet‐only simulations for an additional 10 kyr beyond the end of the iterative procedure (Figures 7g and 7h). It is thus not apparent that the iterative procedure could have been terminated earlier and replaced with the more computationally efficient ice sheet‐only configuration, as both the overall GrIS geometry and internal temperature structure depend on the length of the simulation. Ending an ice sheet‐only simulation too late will result in an ice sheet state that is not consistent with the coupled model climate and may introduce spurious transients in the ice sheet and in the other model components when the ice sheet is reintroduced into a coupled model setting. This defeats the purpose of resorting to the computationally cheaper ice sheet‐only simulation, as the fully coupled model would then have to be run longer to find a new equilibrium state that is free from these transients. As a result of these comparisons, we suggest that maintaining the full iterative approach throughout the spin‐up is more appropriate and robust, than resorting to a (more computationally efficient) standalone spin‐up method partway through the overall procedure.

## 6 Discussion

In this manuscript we have described and demonstrated a computationally tractable, iterative procedure for spinning up a contemporary, highly complex ESM. The utility of the method is illustrated by spinning up the preindustrial climate in CESM2 (Danabasoglu et al., 2020), using a model configuration that includes an interactive GrIS. With this method, this final coupled ice sheet/ESM state is achieved for a computational cost that is very significantly (up to an order of magnitude) less than other ice sheet/Earth system spin‐up methods, and of the same order of magnitude as traditional non‐ice sheet‐enabled ESM spin‐up/equilibration methods. This outcome is the primary result of this model development‐specific study and demonstrates that ice sheet‐enabled ESMs can be economically conditioned to preindustrial climate states in preparation for historical and future transient simulations in support of sea level rise projections. Beyond this specific application, which we deem critical to the practical use of coupled ice sheet‐ESMs, the method described here is potentially general in nature and therefore may also apply to other ESM configurations. For example, the method may be a viable alternative approach to traditional “brute force” methods for spinning up the deep ocean circulation in coupled models and may also be applicable for rapidly spinning up ESM‐simulated coupled climate states for paleo‐climate applications.

Following CMIP6 guidelines (e.g., Eyring et al., 2016), the iterative spin‐up procedure described here consists of a single model integration with perpetual 1850 CE forcing. We emphasize that parameter perturbation and ensemble simulations are not typically implemented when spinning up contemporary, high‐complexity ESMs (although the method could certainly be applied to reduce the computational burden of such efforts, where multiple, parallel spin‐up simulations are required). While the final spun‐up model state is targeted to be in statistical equilibrium, the ice sheet component is not expected to be in perfect balance with the simulated preindustrial climate, due to a retained memory of past glacial conditions. The spun‐up GrIS is thus (correctly) in a nonequilibrium state, consistent with prior climate history. This intentional retention of climate conditions inherited from initial simulation conditions is at general odds with typical ESM spin‐ups, which aim for complete loss of this signal by the end of the spin‐up simulation.

In the approach described here, the modeled coupled climate system is spun up by alternating between a fully coupled model configuration and a computationally cheaper configuration where the atmospheric component is replaced by prescribed boundary condition data model. In order to converge to the results of an equivalent free‐running fully coupled model integration, simulation segments with the data atmosphere are kept short enough to prevent unrealistic climate drift, but long enough to capitalize on the greater throughput and lower cost relative to the fully coupled configuration; see Tables 1 and 2. With this approach, the cost of the atmospheric model (which accounts for almost 70% of the total cost of CESM2 at the standard 1° resolution) is greatly reduced, with no significant divergence of final spun‐up model state.

The computational requirements for the iterative procedure are an order of magnitude smaller than for an equivalent application with a fully coupled model configuration. For practical context, the resource requirements for a synchronously coupled ice sheet/Earth system simulation of the same length would be a substantial fraction of the annual computing resources available to entire national‐level modeling centers (∼25 M core hours; Table 2) and are thus not feasible to carry out without sacrificing other key simulations. The cost of the iterative procedure is also around 30% lower than a traditional asynchronous ice sheet/climate coupling commonly adopted for long integrations with ice sheet/climate models (e.g., Ridley et al., 2005); see Table 2. Our analysis of computational savings is based on an iterative approach that involves 35 years of coupled model simulation, followed by 150 years of simulation with a data atmosphere. We determined that this gives a reasonable balance between resource savings and an acceptable final, internally consistent coupled climate state. Nonetheless, further cost savings could likely be obtained with adjustments to the simulation procedure. Irregardless of further methods tuning, we highlight that the overall procedure provides a practical method for very notably reducing the computational expense of developing a self‐consistent coupled preindustrial ice sheet/Earth system state, to the point where this exercise is now manageable within existing computational capacities of individual modeling group. This result is significant, because achieving this state is a key link in broader efforts to develop global sea level rise projections for adaptation planning.

While we believe our method to be unique in its implementation and efficiency, it is not the first to tackle the challenge of reducing the computation expense of coupled ice sheet/Earth system spin‐up simulations. For example, in a recent study, Ziemen et al. (2019) introduced a novel acceleration technique for running ECHAM5 with interactive ice sheets over glacial timescales. Similar to our methodology, they used a periodic synchronous coupling and achieved substantial computational savings by recycling atmospheric data over much of the simulation. Their implementation, however, is notably different from ours, which makes for an interesting comparison. They effectively implemented a 1/10/100 atmosphere/climate/ice sheet coupling, where the coupled model was run for a single year every 10 ocean‐land‐sea ice model years, and every 100 ice sheet model years. Between these segments, the data atmosphere provided atmospheric forcing stored from the previous five coupled model years. This means that the first and last years of the atmospheric forcing data are separated by as much as 50 years.

This approach was designed to reduce overall computational expenses. However, similar to all accelerated modeling implementations, it may introduce inconsistencies that influence overall climate evolution. For example, several major modes of variability (e.g., ENSO and NAO) typically occur with a periodicity of up to subdecadal timescales. Hence, there is a possibility that sampling every 10 model years could result in aliasing of major climate modes in the atmosphere data archive. Moreover, running the coupled model for only 1 year after advancing the overall climate/ice sheet state by 10/100 years (respectively) could introduce inconsistencies in the long‐term climate evolution. Specifically, although the atmosphere is quick to adapt to changes in planetary boundary conditions, it can still take several months for full adjustments to abrupt changes in surface conditions, notably the ice sheet topography.

In contrast, the philosophy adopted when designing our simulation procedure was to (1) capture the model's internal climate variability by prescribing high‐frequency atmospheric data over a standard climatological period of three decades; (2) regain internal consistency (i.e., update the data archive) with reasonable periodicity by running relatively short data atmosphere segments (although long compared to Ziemen et al., 2019); and (3) suppress “shocks” in the climate system when transitioning between the coupled and data atmosphere configurations, by running the coupled model for 5 years before saving data to the forcing archive.

It is entirely possible that none of these concerns are major issues for the simulations described in Ziemen et al. (2019), and we concede that some of the techniques we have used may not be strictly necessary for the goal of generating a spun‐up climate/ice sheet state similar to that obtained from a coupled model with a synchronous climate/ice sheet coupling. Nevertheless, based on the above comparison, we suggest general caution when applying asynchronous and periodic synchronous simulation techniques, to avoid the risk of unintended model behavior.

The simulations described here have their own shortcomings. Most prominently, the equilibrium ice sheet is 10% to 20% larger than modern observations. However, this is primarily a result of biases in the simulated GrIS SMB, and not a product of the spin‐up procedure itself. Thus, this final bias should not be interpreted as a failure of the spin‐up procedure. Notably, van Kampenhout et al. (2019) recently showed that the relatively coarse (nominal 1°) horizontal resolution of the atmosphere model is linked to biases in the SMB, by demonstrating that regional grid refinement over the ice sheet improves simulation of ice sheet margin precipitation gradients and reduces excessive moisture advection into the ice sheet interior. With respect to the spin‐up procedure, we note that simulations with variable‐resolution grids will likely be more common in the future and should be entirely compatible with spin‐up approaches such as described here.

The final coupled GrIS and Earth system state achieved via our spin‐up technique can be put in context with other results from the ISMIP6 (Nowicki et al., 2016) and, in particular, the ISMIP6 initMIP‐Greenland component (Goelzer et al., 2018). Unlike many intercomparison projects, initMIP‐Greenland puts few constraints on how participating models implemented boundary condition forcing, numerical methods, model parameter choices, and physical approximations. Stemming from this loose application of guidelines, of the 35 standalone ice sheet simulations submitted by 17 participating groups, 22 simulations used similar initialization techniques that, like the procedure presented here, were based on a freely evolving ice sheet. The remaining participating models/simulations used data assimilation of surface topography and/or surface velocities to develop an initial state in close similarity to the observed GrIS. All the freely evolving initMIP‐Greenland simulations overestimated ice sheet volume (ranging from 2.98 to 3.41 M km^3^ (1–16%), compared to the observed 2.95 M km^3^ volume Morlighem et al., 2014), and ice sheet area (ranging from 1.66 to 2.10 M km^2^ (0.6–27%), compared to the observed 1.65 M km^2^). Of these 22 simulations, five ended with a larger volume than from our iterative procedure (ranging from 0.05 to 0.20 M km^3^ larger, about 2.5–10%), and five simulations ended with a larger GrIS area (differences up to 0.10 M km^2^, about 3%). Similar to our final ice sheet state, eight of the 22 freely evolving simulations showed partial or full glaciation of northern Greenland tundra. This comparison, however, is somewhat biased, as several participating models used constant SMB forcing from high‐resolution regional climate simulations, themselves constrained by reanalysis data. Regional climate models can better represent topography and local SMB gradients than comparatively coarser resolution ESMs, which are also not numerically constrained to match regional observed climate conditions. It is thus reassuring that CESM2‐CISM2 falls within the range of these models, despite being a global ESM.

In previous work with one‐way‐coupled CESM1‐CISM1 (Lipscomb et al., 2013), GrIS initialization was carried out with an ensemble of 100 ice sheet‐only simulations via Latin Hypercube sampling of key ice sheet model parameters (similar to Stone et al., 2010). In all ensemble members, the total ice sheet volume and area were overestimated compared with observations. Compared with the iterative procedure described here, ensemble ice volumes were generally larger (difference ranging from −0.05 to +0.65 M km^3^, about −1.5–20%) while ensemble ice areas were generally smaller (−0.03 M km^2^ in all simulations, about −1.5%), with most of the excess volumes added to the northern and southern margins, as in the present case.

Earlier work on coupled climate/ice sheet modeling spun up the ice sheet through the last two glacial cycles using perturbed precipitation and temperature fields (e.g., Ridley et al., 2005). These coupled simulations also overestimated the ice sheet volume compared to the present‐day observed GrIS, but by 0.25 M km^3^ (8%) less than the results presented here, mainly because the SMB was much lower (285 vs. 591 Gt/year). Vizcaino et al. (2015) used a two‐step spin‐up procedure, where the ice sheet was initially forced with uncorrected precipitation and temperature fields until 9 ka and then bidirectionally coupled to a climate model with an energy‐balance‐based SMB calculation. This produced an initial GrIS that exceeded the observed ice sheet in both area and volume, though with respect to the current study the area was 0.05 M km^2^ (2%) smaller, and the volume was 0.11 M km^3^ (3%) smaller. Altogether, the spun‐up GrIS state from our iterative procedure is well within the range of previous ice sheet model spin‐ups.

These comparisons indicate that the final result of the iterative spin‐up procedure displays biases that are similar in nature to previous efforts. This observation no way invalidates the iterative spin‐up procedure, because these biases relate instead to intrinsic CESM2 climate and/or CISM2 ice sheet biases. Nonetheless, documentation of the bias is important, as it frames future bias reduction efforts (likely using the spin‐up procedure to cheapen the related computation cost).

## 7 Conclusions

In this study we have described and demonstrated a computationally efficient iterative procedure for spinning up coupled ice sheet/ESMs. We test its applicability by spinning up the preindustrial climate in CESM2, including a two‐way interactive ice sheet model (CISM2) over Greenland. We summarize our methods and conclusions as follows:

(i) The iterative spin‐up procedure alternates between a fully coupled and a computationally cheaper configuration where the atmospheric component is replaced by a data model. By periodically regenerating the atmospheric forcing, the data atmosphere remains adequately constrained to respect coupled conditions, ensuring that the broader coupled model state does not drift unrealistically.
(ii) The simulated climate at the end of the spin‐up procedure is similar to the preindustrial control climate from a 1,200‐year fully coupled spin‐up simulation with a prescribed GrIS, indicating that the climate in our iterative procedure converges toward a climate state that is consistent with a traditional spin‐up approach despite a significant computational savings.
(iii) The iterative method is an order of magnitude faster and cheaper than a traditional “brute force” synchronous spin‐up method, and significantly faster and cheaper than other accelerated/asynchronous spin‐up methods.
(iv) The iterative method presented here could enable faster and cheaper spin‐ups for computationally expensive coupled climate and ESMs, with or without interactive ice sheets.

The preindustrial ice sheet/Earth system state achieved via the new spin‐up method will provide initial conditions for transient simulations with CESM2‐CISM2 in support of ISMIP6 coupled historical and future simulations (e.g., Muntjewerf et al., 2020). Further results presented in future ISMIP6, CMIP6, and subsequent studies with an interactive GrIS thus will rely substantially on the techniques developed and presented here.

## Code Availability Statement

The simulations in this study were produced with the publically released version of CESM2.1.1, which is open source software (freely available at http://www.cesm.ucar.edu/).

## Data Availability

Data presented in this work are publicly available on the Earth System Grid (account registration is required: https://www.earthsystemgrid.org/dataset/ucar.cgd.cesm2.CESM21-CISM2-JG-BG.html).
